# Supplementary material for: Two-dimensional electronic spectroscopy of bacteriochlorophyll a with synchronized dual mode-locked lasers
Source: Nat Commun. 2020 Nov 27;11:6029. doi: 10.1038/s41467-020-19912-5 (PMC7699642; doi:10.1038/s41467-020-19912-5)
Supplement: Supplementary file 1 — Supplementary Information [file 41467_2020_19912_MOESM1_ESM.pdf]

Supplementary Information

Two-dimensional electronic spectroscopy of bacteriochlorophyll *a* with synchronized dual mode-locked lasers

JunWoo Kim,[1] Jonggu Jeon,[1] Tai Hyun Yoon,[1,2*] and Minhaeng Cho[1,3*]

[1]*Center for Molecular Spectroscopy and Dynamics, Institute for Basic Science (IBS), Seoul 02841, Republic of Korea*

[2]*Department of Physics, Korea University, Seoul 02841, Republic of Korea*

[3]*Deparment of Chemistry, Korea University, Seoul 02841, Republic of Korea*

*Electronic mails: mcho@korea.ac.kr (MC) or thyoon@korea.ac.kr (THY)

Supplementary Note 1: Experimental methods

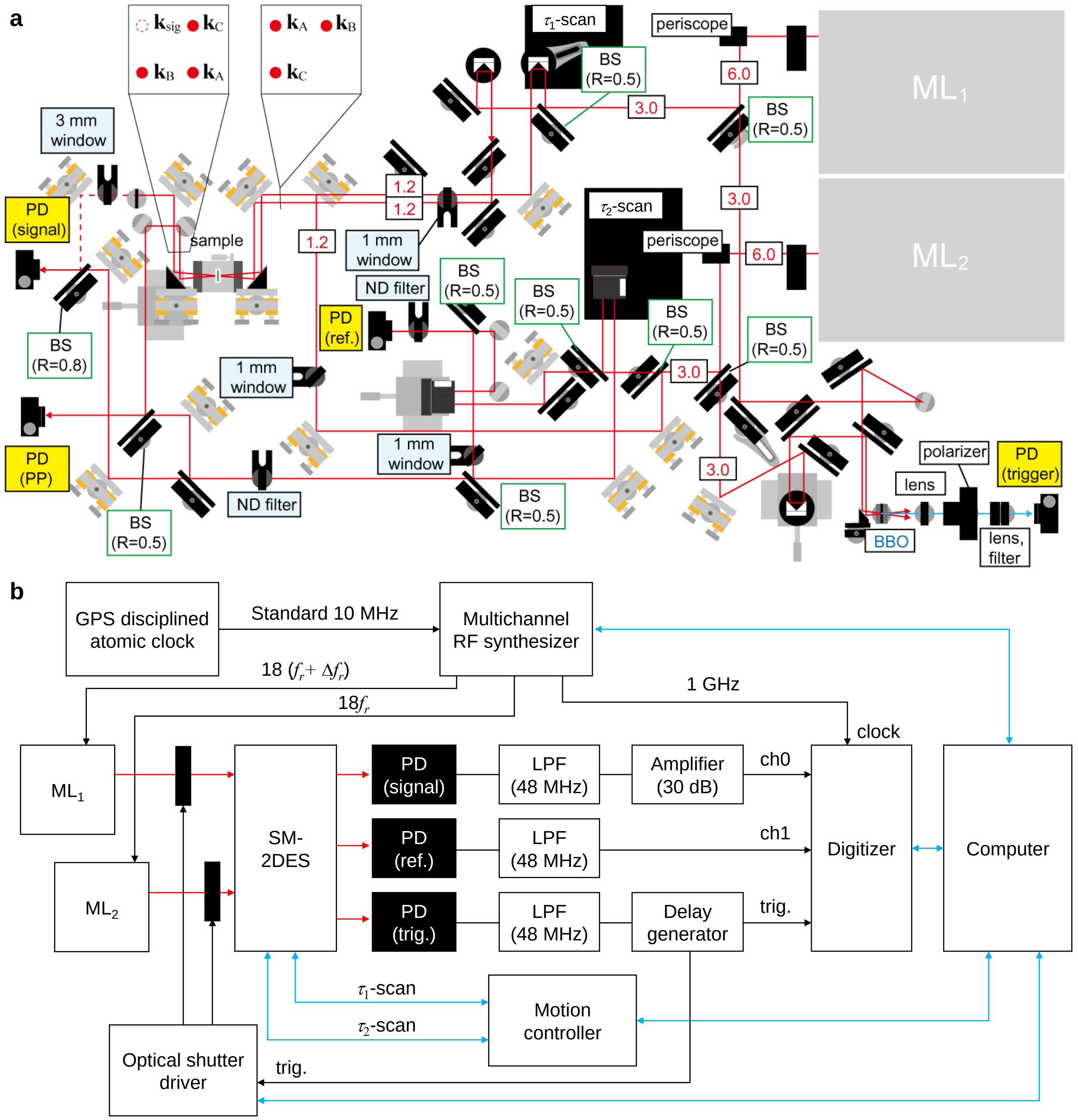

Supplementary Figure 1| Experimental setup of SM-2DES. a. Optical layout of SM-2DES. The red numbers on beamlines correspond to single pulse energies (in nJ) measured at these specific positions. The two boxes on the top-left corner show the front views of the optical beamlines before (right) and after (left) the sample. ML, repetition rate-stabilized mode-locked laser; BS, beam splitter; BBO, beta-barium borate crystal; PD, photodetector; ND filter, neutral density filter; PP: pump-probe. **b**. Schematic diagram of the SM-2DES instrumentation. Black (blue) arrows indicate unidirectional (bidirectional) electronic connections, while red arrows represent optical beam propagations. LPF, low-pass filter.

Supplementary Figure 1a depicts the laboratory optical layout of our SM-2DES system. Two 7-fs Ti:Sapphire mode-locked lasers (MLs; Rainbow 2, Femtolasers) were used as the light sources. The repetition rates of the MLs were phase-locked to the 18th harmonics of 80 MHz + Δf_r (ML_1) and 80 MHz (ML_2), respectively. Here, the ground state recovery time, which is usually the lifetime of the excited state, should be shorter than the pulse repetition period, $1/f_r$. Otherwise, undesired multi-photon processes via a train of pulses from high repetition rate lasers could make the SM-2DES signal obscure and the interpretation difficult. Δf_r was controlled to be either 38.4 Hz or 3.2 kHz for femtosecond and nanosecond SM-2DES measurements, respectively, of the BChla solution. In the present work, we considered these Δf_r values, 38.4 Hz and 3.2 kHz, to investigate two different relaxation dynamics that are the vibrational dephasing processes of vibronically coupled modes and the population relaxation of electronically excited states, respectively. The vibrational coherences dephase in a few picoseconds at room temperature while the population relaxation of electronically excited state takes place on the order of nanoseconds. To investigate them separately and more effectively, we performed SM-2DES measurements in two different time-scan modes, i.e., slow-scan mode with $\Delta T_w = 6$ fs ($\Delta f_r = 38.4$ Hz) for monitoring the underdamped oscillations of vibronically coupled modes and fast-scan mode with $\Delta T_w = 500$ fs ($\Delta f_r = 3.2$ kHz) for probing slow population relaxation processes. Half of the laser output was used for an optical trigger generation. For femtosecond-resolved measurements with $\Delta f_r = 38.4$ Hz, the separated beams for the optical trigger were non-collinearly focused at a 100-μm thick BBO crystal (CASTECH) with a parabolic mirror (f = 25 mm). The sum-frequency-generation signal produced by the temporal and spatial overlap of pulses from the two MLs is used as an optical trigger. For nanosecond measurements with $\Delta f_r = 3.2$ kHz, a 10-mm thick fused silica window was placed right before the parabolic mirror, and the 100-μm thick BBO crystal was replaced by a 1-mm thick BBO crystal. The pulse duration of the optical trigger must be larger than ΔT_w to avoid missing trigger pulse due to time-jitter.[1] The manual stage in the trigger line (the right-bottom side in Supplementary Figure 1a) sets the time-zero of the waiting time ($T_w = 0$).

Each pulse from ML_1 was further separated into two, and they are directed in the direction of $\mathbf{k}_A$ and $\mathbf{k}_B$. The optical time delay between them, denoted as τ_1, was controlled by a motorized stage (XMS-160, Newport). The pulses from ML_2 propagate in the direction of $\mathbf{k}_C$. All of them were focused by a parabolic mirror with $f = 100$ mm and are collimated by another mirror with $f =$

100 mm after the sample. Single pulse energy of each beam was measured to be about 1.2 nJ right before the sample. In the nanosecond SM-2DES measurements, the probe pulse energy was deliberately reduced to 0.6 nJ to minimize the undesired contributions from higher-order nonlinear optical signals and thermal heating effects. (see Supplementary Note 4 for details on the effect of local heating effect) The signal beam is generated along the direction of $\mathbf{k}_{sig} = -\mathbf{k}_A + \mathbf{k}_B + \mathbf{k}_C$ as a result of the third-order (in the electric fields) interaction at the sample. The front views of the four beams before and after the sample can be found in the boxes on the top-left corner of Supplementary Figure 1a. The signal electric field was combined with a local oscillator from ML_2, and the total intensity was detected at a PD (signal PD in Supplementary Figure 1a). The time delay between the signal and local oscillator denoted as τ_2 was controlled by another motorized stage (XMS-160, Newport). Small portions of the local oscillator were separated before and after the τ_2-scanning stage and used to construct a Mach-Zehnder interferometer used for phase referencing of each τ_2-scan. The time-resolution of the experiment was estimated by means of measuring the transient grating signal from a BBO crystal that was placed at the sample position. A thin BBO crystal (thickness is less than 20 μm) is usually appropriate for characterizing the pulse width of a-few-fs light sources because of the broad sum-frequency-generation (SFG) bandwidth of such a thin nonlinear crystal. However, we found that the intensity of the sum-frequency field generated by ML_1 and ML_2 with a 20-μm-thick BBO crystal at the sample position is too weak to compress the optical pulse widths of the MLs efficiently. Therefore, we used a 100-μm-thick BBO crystal with a high SFG efficiency to measure the cross-correlation between ML_1 and ML_2. The estimated time resolution is 12.5 fs (full-width-at-half-maximum), even though the ideal time resolution for the present 2DES experiment when the pulse duration time is 7.0 fs would be 10.0 fs. This time resolution enables us to measure the coherent vibrational spectrum obtained from the Fourier transform of the SM-2DES data with respect to the waiting time. The upper limit of the measurable vibrational frequencies is 2665 cm^{-1} because the corresponding oscillation period is 12.5 fs.

SM-2DES is advantageous for removing multiple artifacts occurring near $T_w = 0$. Firstly, it is nearly free from the solvent response and higher-order electronic response from the optical sample due to the low pulse energies (~1 nJ) involved in the experiment. The contribution from light scatterings by impurities, e.g., aggregates of solute molecules in the sample, does not affect the 2D spectra, because a full T_w-scan takes a time of $1/\Delta f_r$ while τ_1 and τ_2 are fixed at constants. Thus,

the contribution from scatterings could be removed during the double Fourier transformations of $I(\tau_1,T_w,\tau_2)$ with respect to τ_1 and τ_2.

The data recording schematics of our SM-2DES is shown in Supplementary Figure 1b. A multichannel RF synthesizer (HS9008, Holzworth) phase-locked to a standard frequency provides reference frequencies to the two MLs. A digitizer with the bandwidth of 90 MHz (M3i.4861-exp, Spectrum) is synchronized to the standard frequency, and its sampling rate was set to be the same as the repetition rate of ML_2 (80 MHz). The trigger signal measured at the trigger PD passes a 48-MHz low-pass filter and enters a delay generator (DG645, Stanford Research Systems) as a trigger source. The delay generator triggers data acquisition and controls optical shutters. Two optical shutters are placed right after the MLs and are used to minimize the thermal damage on the sample. For femtosecond-resolved measurements with Δf_r = 38.4 Hz, the shutters are synchronized to the optical trigger, so it is opened only at the detection time window. The same gated sampling cannot be achieved when we set the repetition frequency detuning factor Δf_r to be 3.2 kHz because the T_w-scan rate (Δf_r = 3.2 kHz) was faster than the bandwidth of the optical shutters (100 Hz). Instead, the shutters were opened when τ_2 was being scanned.

The τ_1- and τ_2-scans were controlled by a motion controller (XPS-RL, Newport) with two high-resolution drivers (DRV-02L, Newport). The ranges of τ_1- and τ_2-scans were set large enough to fully record the interferogram envelope arising in the data (135 fs in optical time-delay). τ_2 was scanned continuously with a constant velocity (v_2), which was stabilized with the 8 kHz servo rate by the motion controller. v_2 was set by considering the effective increment of τ_2 ($\Delta\tau_2$) based on the relation

$$\Delta\tau_2 = \frac{2n_{air}v_2}{c\Delta f_r}, \quad (1)$$

where n_{air} and c are the refractive index of air and the speed of light, respectively. For femtosecond-resolved measurements with Δf_r = 38.4 Hz, $\Delta\tau_2$ was set as large as possible, satisfying the Nyquist sampling condition, to achieve the highest data acquisition speed. The final $\Delta\tau_2$ value was 1.04 fs, which corresponds to v_2 = 5.0 μm/s. For nanosecond measurements with Δf_r = 3.2 kHz, setting a large $\Delta\tau_2$ accompanying a high v_2 was inefficient because about 200 ms was required to stabilize the velocity at high v_2. Thus, the scan range became unnecessarily large to get larger $\Delta\tau_2$ at Δf_r = 3.2 kHz. The optimized $\Delta\tau_2$ was 0.21 fs (v_2 = 50 μm/s) for the nanosecond-SM-2DES

measurements. Finally, for every τ_2-scan, the step of the mechanical delay stage used for τ_1-scan was set to be 0.14 μm, which corresponds to 0.93 fs in the time domain. The interferometric pump-probe (IPP) data was recorded with the same instrument but without τ_1-scan at the PD (see Supplementary Figure 1a).[1] The pump-probe data provides an absolute phase reference to the 2DES data (see Supplementary Note 3 for details). It should be mentioned that the size of a single τ_2-scan data set ($N_{\tau 2} \times N_{Tw}$) is determined by the onboard memory of the fast digitizer, which is 2 GB with the resolution of 16 bit in the present work. Here, $N_{\tau 2}$, and N_{Tw} are the numbers of τ_2 and T_w data points, respectively.

Although the absorption coefficient of BChla ($\varepsilon_{\mathrm{BChla}} \cong 5\times10^5$ M^{-1}cm^{-1}) is comparable to that of IR125 ($\varepsilon_{\mathrm{IR125}} \cong 10\times10^5$ M^{-1}cm^{-1}), the experimentally measured 2DES signal of BChla is found to be weaker than that of IR125. This discrepancy could result from the formation of a long-lived triplet state BChla.[2] Due to the high repetition rate of the pump ML used here, the population of the triplet state could be accumulated and reaches a quasi-equilibrium state during the course of measurements, which reduces the net population of the electronic ground state. Therefore, the SM-2DES signal amplitude could depend on the repetition rate of the ML. Currently, the effect of a high repetition frequency excitation beam is under investigation theoretically by numerically solving the kinetic equations involved with long-lived states and calculating the resulting 2DES signals upon varying repetition frequencies. (see Supplementary Note 3 for details)

The data acquisition time for a complete set of SM-2DES data is primarily determined by the velocity of τ_2−scanning translation stage v_2 and the data transportation time from the digitizer to the computer storage. For femtosecond-resolved measurements with Δf_r = 38.4 Hz, a single τ_2−scan took about 5 s while the data acquisition took tens of milliseconds. In contrast, the data transportation time was the rate-determining process when we carried out nanosecond-SM-2DES measurements with Δf_r = 3.2 kHz, where it took about 10 s in the present experiments. Thus, the total data acquisition times for femtosecond-resolved and nanosecond-SM-2DES with Δf_r = 38.4 Hz and 3.2 kHz, respectively, were 7 min and 4 min. To improve statistics, we repeated such measurements 32 times for bacteriochlorophyll *a*, and report the average here.

Supplementary Note 2: Theoretical description of SM-2DES

In molecular spectroscopy, chromophores interact with incident electric fields and, within the electric dipole approximation, the radiation-matter interaction Hamiltonian can be written as

$$H_{\text{int}}(t) = -\boldsymbol{\mu}\cdot\boldsymbol{\mathcal{E}}(\mathbf{r},t)\,, \tag{2}$$

where $\boldsymbol{\mu}$ is the electric dipole operator and $\boldsymbol{\mathcal{E}}(\mathbf{r},t)$ is the superposition of the electric fields used. Hereafter, we treat all the electric field quantities as scalar representing a common direction of polarization. The total Hamiltonian of the system is the sum of the system Hamiltonian H_0 in the absence of radiation and radiation-matter interaction Hamiltonian. The electric field of the pulse train j can be written as

$$\mathcal{E}_j(\mathbf{r},t) = \frac{1}{2}\left[E_j(\mathbf{r},t) + \text{c.c.}\right], \tag{3}$$

where the complex electric fields are given by[3]

$$E_j(\mathbf{r},t) = \sum_{n=-\infty}^{\infty} A_j(t-n\Delta T_j)e^{i\left[\mathbf{k}_j(\omega_{cj})\cdot\mathbf{r}-\omega_{cj}(t-n\Delta T_j)-n\Delta\phi_{ceo,j}\right]} \tag{4}$$

in terms of the time interval ΔT_j between neighboring pulses of the jth optical frequency comb (OFC), the carrier frequency ω_{cj}, and the carrier-envelope-phase shift $\Delta\phi_{ceo,j}$ between successive pulses.

For a coherent and periodic pulse train, the relative phase between two consecutive pulses in Supplementary Equation 4 must be an integer multiple of 2π, and this imposes the following condition on the carrier frequency of the jth OFC laser,[3,4]

$$\omega_{cj} = n_{cj}\omega_{rj} + \omega_{\text{ceo},j}\,, \tag{5}$$

where n_{cj} is the integer mode number characterizing the carrier frequency of the jth OFC. Here, we introduced the following definitions:

$$\begin{aligned} \omega_{rj} &= 2\pi/\Delta T_j\,, \\ \omega_{\text{ceo},j} &= \Delta\phi_{\text{ceo},j}\,\omega_{rj}/(2\pi). \end{aligned} \tag{6}$$

Then, $E_j(\mathbf{r},t)$ can be written as[4]

$$\begin{aligned} E_j(\mathbf{r},t) &= e^{i\mathbf{k}_j(\omega_{cj})\cdot\mathbf{r}-i\omega_{cj}t}\sum_{n=-\infty}^{\infty} A_j(t-n\Delta T_j) \\ &= e^{i\mathbf{k}_j(\omega_{cj})\cdot\mathbf{r}-i\omega_{cj}t}\sum_{n=-\infty}^{\infty} A_{nj}e^{-in\omega_{rj}t} \end{aligned}, \tag{7}$$

where A_{nj} is the Fourier expansion coefficient of the pulse envelop function defined as

$$A_{nj} = (1/\Delta T_j)\int_{-\infty}^{\infty} A_j(t)e^{2\pi int/\Delta T_j}dt. \tag{8}$$

The OFC spectrum obtained by Fourier transforming Supplementary Equation 7 can be shown to have peaks at the following frequencies

$$\omega = \omega_{cj} + n\omega_{rj} = \omega_{\text{ceo},j} + m\omega_{rj} \quad (m,n = \text{integers}), \tag{9}$$

where the second equality comes from Supplementary Equation 5. For later use, we introduce the differences in the repetition and carrier frequencies of the two OFC fields as

$$\begin{aligned} \delta\omega_r &= \omega_{r1} - \omega_{r2}, \\ \delta\omega_c &= \omega_{c1} - \omega_{c2}. \end{aligned} \tag{10}$$

In the present experiment, the sample, dye molecules in solution, interacts with three sets of pulse trains: (i) OFC_1 in the $\mathbf{k}_1$ direction, (ii) OFC_1 delayed by τ_1 (which is much smaller than the repetition period of OFC_1 pulse train) from the original OFC_1 field and redirected in the $\mathbf{k}_2$ direction, (iii) OFC_2 in the $\mathbf{k}_3$ direction. The waiting time T_w between the second and third interactions is scanned automatically by the pulse trains of OFC_1 and OFC_2 that have slightly different pulse repetition rates by $\delta\omega_r$. The signal in the $-\mathbf{k}_1 + \mathbf{k}_2 + \mathbf{k}_3$ direction is heterodyne-detected with the OFC_2 field as the local oscillator (LO), which is redirected in the signal direction and is delayed by τ_2 (which is less than the repetition period of OFC_2 pulse train) from the third interaction. Note that τ_1 and τ_2 are explicitly controlled and scanned by, e.g., mechanical delay stages, but the waiting time T_w is determined implicitly by the repetition frequencies of the two OFCs and their offset $\delta\omega_r$. Experimentally, the time origin for T_w is set with an optical trigger signal produced when the sum-frequency-generation field produced by the overlap of the OFC_1 and OFC_2 pulses in a nonlinear crystal is detected.

The total electric field incident on the sample is the superposition of the three sets of pulse trains, i.e., $\mathcal{E}(\mathbf{r},t) = \mathcal{E}_1(\mathbf{r},t) + \mathcal{E}_2(\mathbf{r},t) + \mathcal{E}_3(\mathbf{r},t)$, where $\mathcal{E}_1(\mathbf{r},t)$ and $\mathcal{E}_2(\mathbf{r},t)$ are derived from the OFC_1 field and $\mathcal{E}_3(\mathbf{r},t)$ from the OFC_2 field. The three complex electric fields can then be written as follows

$$E_1(\mathbf{r},t) = e^{i\mathbf{k}_1\cdot\mathbf{r}-i\omega_{c1}(t+\tau_1)}\sum_{n=-\infty}^{\infty} A_{n1}e^{-in\omega_{r1}(t+\tau_1)},$$

$$E_2(\mathbf{r},t) = e^{i\mathbf{k}_2\cdot\mathbf{r}-i\omega_{c1}t} \sum_{n=-\infty}^{\infty} A_{n1} e^{-in\omega_{r1}t}, \tag{11}$$

$$E_3(\mathbf{r},t) = e^{i\mathbf{k}_3\cdot\mathbf{r}-i\omega_{c2}t} \sum_{n=-\infty}^{\infty} A_{n2} e^{-in\omega_{r2}t}.$$

Note that $t = 0$ corresponds to zero waiting time T_w, that is, zero time gap between the second and third field-matter interactions for infinitesimally narrow pulses. The third-order polarization generated by this electric field is given by

$$P^{(3)}(\mathbf{r},t) = \int_0^\infty dt_3 \int_0^\infty dt_2 \int_0^\infty dt_1 S^{(3)}(t_3,t_2,t_1)\mathcal{E}(\mathbf{r},t-t_3)\mathcal{E}(\mathbf{r},t-t_3-t_2)\mathcal{E}(\mathbf{r},t-t_3-t_2-t_1), \tag{12}$$

where $S^{(3)}(t_3,t_2,t_1)$ is the third-order response function. We then detect the signal along the direction of $\mathbf{k}_s = -\mathbf{k}_1 + \mathbf{k}_2 + \mathbf{k}_3$. The third-order polarization generating this signal can be written as $P^{(3)}(\mathbf{k}_s,t) = P^{(3)}(t)\exp(i\,\mathbf{k}_s\cdot\mathbf{r})$, which can be expressed as follows:

$$\begin{aligned} P^{(3)}(t) = \frac{1}{8}\int_0^\infty dt_3 \int_0^\infty dt_2 \int_0^\infty dt_1 S^{(3)}(t_3,t_2,t_1) \\ \times\{ E_1^*(t-t_3)E_2(t-t_3-t_2)E_3(t-t_3-t_2-t_1) \\ + E_1^*(t-t_3)E_3(t-t_3-t_2)E_2(t-t_3-t_2-t_1) \\ + E_2(t-t_3)E_1^*(t-t_3-t_2)E_3(t-t_3-t_2-t_1) \\ + E_2(t-t_3)E_3(t-t_3-t_2)E_1^*(t-t_3-t_2-t_1) \\ + E_3(t-t_3)E_1^*(t-t_3-t_2)E_2(t-t_3-t_2-t_1) \\ + E_3(t-t_3)E_2(t-t_3-t_2)E_1^*(t-t_3-t_2-t_1)\} \end{aligned} \;. \tag{13}$$

According to Supplementary Equation 11, within a time span $0 \le t < 2\pi/\omega_r$, E_1 always arrives first at the sample followed by E_2, then by E_3. On the other hand, the interaction times represented by the time arguments in each $E_k(\mathbf{r},t)$ in Supplementary Equation 13 do not necessarily follow this order. For example, the first term in the integrand corresponds to the situation where E_3 interacts first with the sample at time $t-t_3-t_2-t_1$, E_2 interacts next at $t-t_3-t_2$, then E_1 at $t-t_3$, disregarding minor exceptions due to finite τ_1 or τ_2. From this consideration, only the sixth term above is consistent with the time ordering of the pulses, and all the other terms will only contribute to the signal only for very narrow time windows in t_n (n = 1, 2, 3) set by the pulse widths when two or more pulses overlap significantly. Effectively, each of these five terms becomes non-negligible only if one or more of τ_1, τ_2, or implicit waiting time T_w is smaller than the pulse duration time and

becomes a delta-function-like single point contribution in the impulsive limit. In particular, the first, third, and fourth terms in Supplementary Equation 13, where E_3 precedes E_2, would be non-negligible only for small T_w (< pulse duration time), and therefore can be classified as coherent artifacts. In general, for a waiting time longer than the pulse width, these coherent artifacts would contribute negligibly. In the following development, we focus on the sixth term that is consistent with the time ordering of pulses and therefore is expected to make the predominant contribution to the observed signal. A complete theoretical investigation taking into account all six terms and numerical simulations will be reported elsewhere.[5]

Using the following notation for the nth peak frequency of the jth OFC counted from its carrier frequency

$$\omega_j^n = \omega_{cj} + n\omega_{rj}\,, \tag{14}$$

the sixth term in the integrand of Supplementary Equation 13 can be rewritten as

$$\begin{aligned} &E_3(\mathbf{r},t-t_3)E_2(\mathbf{r},t-t_3-t_2)E_1^*(\mathbf{r},t-t_3-t_2-t_1) \\ &= e^{i\mathbf{k}_s\cdot\mathbf{r}} \sum_{q,m,n=-\infty}^{\infty} A_{n1}^* A_{m1} A_{q2} e^{i(\omega_1^n-\omega_1^m-\omega_2^q)t} e^{i\omega_1^n\tau_1} e^{-i(\omega_1^n-\omega_1^m-\omega_2^q)t_3} e^{-i(\omega_1^n-\omega_1^m)t_2} e^{-i\omega_1^n t_1}\,. \end{aligned} \tag{15}$$

The polarization in $\mathbf{k}_s$ direction due to the sixth term is given by

$$\begin{aligned} P^{(3)}(\mathbf{k}_s,t) = \frac{1}{8} e^{i\mathbf{k}_s\cdot\mathbf{r}} &\sum_{q,m,n=-\infty}^{\infty} A_{n1}^* A_{m1} A_{q2} e^{i(\omega_1^n-\omega_1^m-\omega_2^q)t} e^{i\omega_1^n\tau_1} \\ &\times \tilde{S}^{(3)}(-\omega_1^n+\omega_1^m+\omega_2^q, \omega_1^m-\omega_1^n, -\omega_1^n) \end{aligned}\,, \tag{16}$$

where the frequency-domain response function (susceptibility) is defined as

$$\tilde{S}^{(3)}(\omega_3,\omega_2,\omega_1) \equiv \int_0^\infty dt_3 \int_0^\infty dt_2 \int_0^\infty dt_1 S^{(3)}(t_3,t_2,t_1) e^{i(\omega_3 t_3+\omega_2 t_2+\omega_1 t_1)}\,. \tag{17}$$

The electric field generated by the third-order polarization is[6]

$$E^{(3)}(\mathbf{k}_s,t) \propto \frac{i\omega_s}{n(\omega_s)} P^{(3)}(\mathbf{k}_s,t)\,, \tag{18}$$

where $n(\omega_s)$ is the refractive index of the medium at ω_s. When the signal is heterodyne detected with the LO, which is the redirected and delayed OFC_2 field, the LO field can be written as

$$E_{\mathrm{LO}}(\mathbf{r},t) = e^{i\mathbf{k}_s\cdot\mathbf{r}-i\omega_{c2}(t-\tau_2)} \sum_{n=-\infty}^{\infty} A_{n2} e^{-in\omega_{r2}(t-\tau_2)}\,, \tag{19}$$

which is different from the original OFC$_2$ field $E_3(\mathbf{r},t)$ in Supplementary Equation 11 by the delay time τ_2 and the propagation direction $\mathbf{k}_s$. The signal intensity at the detector is then given by the superposition of E_{LO} and the third-order signal field $E^{(3)}$ as follows

$$I(t)=\left|E_{\mathrm{LO}}(\mathbf{k}_s,t)+E^{(3)}(\mathbf{k}_s,t)\right|^2=\left|E_{\mathrm{LO}}(\mathbf{k}_s,t)\right|^2+\left|E^{(3)}(\mathbf{k}_s,t)\right|^2+2\,\mathrm{Re}\left[E_{\mathrm{LO}}^*(\mathbf{k}_s,t)E^{(3)}(\mathbf{k}_s,t)\right]. \quad (20)$$

In the present experiment, the LO intensity is comparable to that of the signal. Note that the first term on the right-hand side of Supplementary Equation 20 does not depend on τ_1, the second term, a homodyne signal intensity, does not depend on τ_2, and only the interference term does depend on both τ_1 and τ_2. Therefore, the first two terms can be easily removed by Fourier transforming the measured interferogram intensity with respect to τ_1 and τ_2. From Supplementary Equations 16-19, the interference signal can be written as

$$\begin{aligned}&2\,\mathrm{Re}\left[E_{\mathrm{LO}}^*(\mathbf{k}_s,t)E^{(3)}(\mathbf{k}_s,t)\right]\propto 2\,\mathrm{Im}[E_{\mathrm{LO}}^*(\mathbf{k}_s,t)P^{(3)}(\mathbf{k}_s,t)]\\&=\frac{1}{4}\mathrm{Im}\left[\sum_{p,q,m,n=-\infty}^{\infty}A_{p2}^*A_{n1}^*A_{m1}A_{q2}\tilde{S}^{(3)}(-\omega_1^n+\omega_1^m+\omega_2^q,\omega_1^m-\omega_1^n,-\omega_1^n)e^{i(\omega_2^p+\omega_1^n-\omega_1^m-\omega_2^q)t}e^{i\omega_1^n\tau_1}e^{-i\omega_2^p\tau_2}\right].\end{aligned} \quad (21)$$

The frequency factor associated with the measurement time t in this equation can be rewritten as

$$\omega_2^p+\omega_1^n-\omega_1^m-\omega_2^q=-(m-n)\delta\omega_r+(p-q-m+n)\omega_{r2}. \quad (22)$$

In the experiment, only the slowly-oscillating interference terms in the RF domain are selectively detected with a low-pass filter or slow-response detector. Under this condition, the terms with non-zero coefficients of ω_{r2} vanish due to the relation $\omega_{r2}\approx 10^6\delta\omega_r$, and only the terms with the following values of p and associated frequency factors survive when detecting the signal in time t

$$p=q+m-n,\quad \omega_2^p+\omega_1^n-\omega_1^m-\omega_2^q=-(m-n)\delta\omega_r. \quad (23)$$

The heterodyne-detected signal in Supplementary Equation 21 can then be written as

$$\begin{aligned}&2\,\mathrm{Re}\left[E_{\mathrm{LO}}^*(\mathbf{k}_s,t)E^{(3)}(\mathbf{k}_s,t)\right]\propto 2\,\mathrm{Im}[E_{\mathrm{LO}}^*(\mathbf{k}_s,t)P^{(3)}(\mathbf{k}_s,t)]\\&=\frac{1}{4}\mathrm{Im}\left[\sum_{q,m,n=-\infty}^{\infty}A_{(q+m-n)2}^*A_{n1}^*A_{m1}A_{q2}\tilde{S}^{(3)}(-\omega_1^n+\omega_1^m+\omega_2^q,\omega_1^m-\omega_1^n,-\omega_1^n)e^{i(n-m)\delta\omega_r t}e^{i\omega_1^n\tau_1}e^{-i\omega_2^{q+m-n}\tau_2}\right].\end{aligned} \quad (24)$$

Redefining the summation indices as $L=n-m$, $M=n$, and $N=q+m-n$, we have

$$2\,\mathrm{Re}\left[E^*_{\mathrm{LO}}(\mathbf{k}_s,t)E^{(3)}(\mathbf{k}_s,t)\right]\propto 2\,\mathrm{Im}[E^*_{\mathrm{LO}}(\mathbf{k}_s,t)P^{(3)}(\mathbf{k}_s,t)]$$
$$=\frac{1}{4}\mathrm{Im}\left[\sum_{L,M,N=-\infty}^{\infty}A^*_{N2}A^*_{M1}A_{(M-L)1}A_{(N+L)2}\tilde{S}^{(3)}(-L\omega_{r1}+\omega_2^{L+N},-L\omega_{r1},-\omega_1^M)e^{iL\delta\omega_r t}e^{i\omega_1^M\tau_1}e^{-i\omega_2^N\tau_2}\right]. \quad (25)$$

We can express this signal in the following form:

$$2\,\mathrm{Re}\left[E^*_{\mathrm{LO}}(\mathbf{k}_s,t)E^{(3)}(\mathbf{k}_s,t)\right]\propto 2\,\mathrm{Im}[E^*_{\mathrm{LO}}(\mathbf{k}_s,t)P^{(3)}(\mathbf{k}_s,t)]$$
$$=\frac{1}{4}\mathrm{Im}\left[\sum_{L,M,N=-\infty}^{\infty}B_{LMN}e^{iL\delta\omega_r t}e^{i\omega_1^M\tau_1}e^{-i\omega_2^N\tau_2}\right] \quad (26)$$

with

$$\begin{aligned}B_{LMN}&=A^*_{N2}A^*_{M1}A_{(M-L)1}A_{(N+L)2}\tilde{S}^{(3)}(-L\omega_{r1}+\omega_2^{L+N},-L\omega_{r1},-\omega_1^M)\\&=A^*_{N2}A^*_{M1}A_{(M-L)1}A_{(N+L)2}\tilde{S}^{(3)}(\omega_{c2}+N\omega_{r2}-L\delta\omega_r,-L\omega_{r1},-\omega_{c1}-M\omega_{r1})\end{aligned}. \quad (27)$$

This equation clearly shows that the signal under consideration that originates from the sixth term in Supplementary Equation 13 represents a rephasing pathway because the two coherence oscillation frequencies in the first and the third arguments of $\tilde{S}^{(3)}$ have opposite signs (disregarding non-zero comb indices). Using this result, we can rewrite the heterodyne-detected signal $I(\tau_2, t, \tau_1)$ as

$$\begin{aligned}I(\tau_2,t,\tau_1)&=2\,\mathrm{Re}\left[E^*_{\mathrm{LO}}(\mathbf{k}_s,t)E^{(3)}(\mathbf{k}_s,t)\right]\\&\propto\mathrm{Im}\left[\sum_{L,M,N=-\infty}^{\infty}B_{LMN}e^{iL\delta\omega_r t}e^{i\omega_1^M\tau_1}e^{-i\omega_2^N\tau_2}\right]\end{aligned}. \quad (28)$$

To explore the relation of this signal with the waiting time T_w, we first introduce a complex function $S(\tau_2, t, \tau_1)$ defined as

$$S(\tau_2,t,\tau_1)=\sum_{L,M,N=-\infty}^{\infty}B_{LMN}e^{iL\delta\omega_r t}e^{i\omega_1^M\tau_1}e^{-i\omega_2^N\tau_2}\ , \quad (29)$$

which is related to the interference signal as $I(t_2, t, t_1)\propto \mathrm{Im}[S(t_2, t, t_1)]$. To a good approximation, the summation over L in this equation can be replaced by the integral over a frequency variable ω that corresponds to $L\delta\omega_r$, and we obtain

$$S(\tau_2,t,\tau_1)=\frac{2\pi}{\delta\omega_r}\sum_{M,N=-\infty}^{\infty}\bar{\bar{B}}_{MN}(-t)e^{i\omega_1^M\tau_1}e^{-i\omega_2^N\tau_2}\ , \quad (30)$$

where $\bar{\bar{B}}_{MN}(t)$ is the inverse Fourier transform of B_{LMN} regarded as a function of ω using the correspondence $\omega \leftrightarrow L\delta\omega_r$

$$\bar{\bar{B}}_{MN}(t) = \frac{1}{2\pi}\int_{-\infty}^{\infty} d\omega B_{(\omega/\delta\omega_r)MN} e^{-i\omega t} . \tag{31}$$

Using Supplementary Equation 27, $\bar{\bar{B}}_{MN}(t)$ can be shown to be related to the response function $\bar{S}^{(3)}(\omega_{c2} + N\omega_{r2}, (\delta\omega_r/\omega_{r1})t, -\omega_{c1} - M\omega_{r1})$, which is a 2D Fourier transform of the time-domain response function $S^{(3)}(t_3, t_2, t_1)$ over t_1 and t_3, as a convolution with the pulse envelope function (see Supplementary Reference [4] for further details). Therefore, the signal detection time t can be interpreted as the waiting time T_w after down-scaling by the factor $\delta\omega_r/\omega_{r1}$. Now, the nonlinear response function that is related to the photon echo spectroscopy is denoted as $R_{echo}(\tau_2, T_w, \tau_1)$, and it is related to $S(\tau_2, t, \tau_1)$ as $R_{echo}(\tau_2, T_w, \tau_1) \propto iS(\tau_2, t, \tau_1)$. The definition of $R_{echo}(\tau_2, T_w, \tau_1)$ is given by a sum of two four-point time-correlation functions of electric dipole operators as (see Chapter 5 in Supplementary Reference 5 for details)

$$\begin{aligned} R_{echo}(t_3, t_2, t_1) =& < \mu(0)\mu(t_1+t_2)\mu(t_1+t_2+t_3)\mu(t_1)\rho(-\infty) > \\ &+ < \mu(0)\mu(t_1)\mu(t_1+t_2+t_3)\mu(t_1+t_2)\rho(-\infty) > , \end{aligned}$$

where $\rho(-\infty)$ is the density matrix at $t = -\infty$ and $\mu(t_1)$ is the electric dipole operator in the interaction picture. Using the correspondence between the waiting time and the measurement time, i.e., $T_w \leftrightarrow (\delta\omega_r/\omega_{r1})t$, we arrive at the following expression

$$I(\tau_2, \Delta t, \tau_1) \propto \mathrm{Re}[\mathbf{E}_{LO}^*(t)\cdot\mathbf{E}_{signal}(t)] \propto \mathrm{Re}[R_{echo}(\tau_2, T_w, \tau_1)] . \tag{32}$$

This equation is, theoretically, the principal result used to interpret the experimentally measured 2DES spectra in the main text.

Supplementary Note 3: Data analysis and phase correction

The raw SM-2DES data ($I(\tau_{2,q},T_{w,m},\tau_{1,n})$) measured and recorded in a series of time-averaged voltages at the signal PD contains the complete three-dimensional time-domain data. Since 2DES is useful for studying the correlation between the excitation and detection frequencies, the three-dimensionally restructured data, $I(\tau_{2,q},T_{w,m},\tau_{1,n})$, in the time domain should be Fourier transformed to $I(\omega_{2,q},T_{w,m},\omega_{1,n})$ in the frequency domain, where ω_1 and ω_2 are the Fourier conjugate frequencies associated with the two delay times τ_1 and τ_2, respectively. However, to obtain the correct 2DES spectra, appropriate time and phase references are needed. The time-zero of waiting time (T_w) was experimentally and automatically calibrated with each optical trigger (Supplementary Note 1). However, it should be noted that the relative positions of interference envelopes for each τ_2-scan cannot be the same because the start times of the data acquisition and the continuous τ_2-delay scan are not synchronized. The phase error caused by the fluctuation of envelope positions was compensated for using a reference interferogram that was measured at the reference PD (see Supplementary Figure 1a). The SM-2DES spectrum obtained by Fourier-transforming the measured time-domain signal with respect to τ_2 becomes

$$I\left(\omega_{2,q'},T_{w,m},\tau_{1,n}\right)=\mathrm{FT}_{\tau_2}\left[I\left(\tau_{2,q},T_{w,m},\tau_{1,n}\right)\right]\exp[-i\Theta_{q',m,n}], \tag{33}$$

$$\text{where}\quad \Theta_{q',m,n}=\arg\left[\mathrm{FT}_{\tau_2}\left[I_{\mathrm{ref}}\left(\tau_{2,q},T_{w,m},\tau_{1,n}\right)\right]\right]. \tag{34}$$

Since the signal intensity $I(\tau_2,T_w,\tau_1)$ and the reference intensity $I_{\mathrm{ref}}(\tau_2,T_w,\tau_1)$ are phase-synchronized during the non-orthogonal T_w- and τ_2-scans in the laboratory time, one can calibrate the phase of the signal with the phase of the reference signal (Supplementary Equation 34). We checked that the reference signal measured with a slow-response digitizer (< 1 MHz) could be of sufficient use for this phase calibration.

When $\mathbf{k}_A$ pulse precedes $\mathbf{k}_B$ pulse, the rephasing contribution to the 2DES signal is dominant, so that the rephasing signal (I_{RE}) corresponds to the raw data $I(\tau_{2,q},T_{w,m},\tau_{1,n})$ when τ_1 is positive, i.e.,

$$I_{RE}\left(\omega_{2,q},T_{w,m},\tau_{1,n}\right)=I\left(\omega_{2,q},T_{w,m},\tau_{1,n}\right) \tag{35}$$

$$\text{with}\quad \tau_n\geq 0. \tag{36}$$

On the other hand, the non-rephasing contribution becomes important when $\mathbf{k}_B$ pulse interacts with the sample earlier than $\mathbf{k}_A$ pulse. In the conventional 2DES experiments, to obtain the non-rephasing signal separately, one of the mechanical translational stages was used to make τ_1 negative. However, here we did not add the stage to our experimental setup for the sake of instrumental simplicity. Instead, the non-rephasing signal (I_{NR}) could be reconstructed with the following interpolation method,

$$I_{NR}\left(\omega_{2,q},T_{w,m},\tau_{1,n}\right)=\left(1-r_n\right)I\left(\omega_{2,q},T_{w,m},\tau_{1,n}\right)+r_nI\left(\omega_{2,q},T_{w,m+1},\tau_{1,n}\right) \quad (37)$$

$$\text{with } r_n=\tau_n/\Delta T_w-\text{Int}\left[\tau_n/\Delta T_w\right],\text{and } \tau_n\le 0\,, \quad (38)$$

where Int[···] rounds the number inside the bracket to the nearest integer. Note that there should be a loss of time-resolution due to this interpolation scheme. However, this effect is really negligible when ΔT_w is sufficiently ultrashort compared to molecular relaxation processes of interest. The subscripts *RE* and *NR* will be omitted hereafter because the following data analyses will be performed for both rephasing and non-rephasing signals.

Finally, $I(\omega_2,T_w,\tau_1)$ is further Fourier transformed to $I(\omega_2,T_w,\omega_1)$ with respect to τ_1 for both rephasing and non-rephasing signals. Due to the high repetition rate (80 MHz) of our synchronized ML system, the probe beam can be additionally diffracted by a slow thermal grating that is produced by repetitive nonradiative relaxations of electronically excited molecules by two non-collinearly propagating pump pulses (see Supplementary Note 4 for more details). The contribution from the transient thermal grating can be removed from the signal as follows:

$$I\left(\omega_{2,q},T_{w,m},\omega_{1,n}\right)=\text{FT}_{\tau_1}\left[I\left(\omega_{2,q},T_{w,m},\tau_{1,n}\right)\right]\cdot\exp\left[-i\theta_{q,n}\right]-R_{q,n}\,, \quad (39)$$

$$\text{with } R_{q,n}\exp\left[i\theta_{q,n}\right]=\left\langle \text{FT}_{\tau_1}\left[I\left(\omega_{2,q},T_{w,m},\tau_{1,n}\right)\right]\right\rangle, \quad (40)$$

where the average in Supplementary Equation 40 was taken by considering signals around T_w = −500 fs. Since the ML_1 and ML_2 pulses overlap in time every 12.5 ns, T_w = – 500 fs means that T_w = 12.5 ns – 500 fs, which is sufficiently long enough to ignore any further electronic and vibrational relaxations, solvation dynamics, and rotational relaxation. Therefore, the experimentally measured signals at such long waiting times are almost constant in waiting time and purely originate from the thermal grating. Therefore, the average amplitude ($R_{q,n}$) and phase ($\theta_{q,n}$) of the thermal grating

signal measured at around $T_w = -500$ fs (Eq. 오류! 참조 원본을 찾을 수 없습니다.) can be used to remove the thermal grating contribution from the raw data (see Eq. 오류! 참조 원본을 찾을 수 없습니다.). The coherent vibrational spectra (Fig. 3e) extracted from the 2DES signals were obtained by the Fourier transform of $I(\omega_1,T_w,\omega_2)$ with respect to T_w, where the slowly decaying components associated with population relaxations of the electronically excited state, which could be fitted to a bi-exponential function, were removed from the raw data, $I(\omega_1,T_w,\omega_2)$, before the Fourier transformation with respect to T_w. We iteratively varied the apodization window to have a high signal-to-noise ratio and to minimize any loss of frequency resolution. We found that 2.5 ps is the optimum width of the apodization window, which corresponds to a frequency resolution of 8 cm^{-1}. Note that the data points at $T_w < 150$ fs were not taken into account when we carried out Fourier transforms of the T_w-dependent 2DES signals, which was needed to remove the contribution from the coherent artifacts and the complexity due to the spectral overlap of GSB and SE terms at very short times ($T_w < 150$ fs).

Hereafter, we focus on the phase-correction procedure. It is well-known that there exist two intrinsic problems in the conventional 2DES technique,[6] which are (i) the difficulty of calibrating the phase of the 2DES signal and (ii) the difficulty of correctly taking into consideration the effects of non-ideal (non-Gaussian) pulse spectra on the experimentally measured 2DES spectra. In most conventional 2DES with boxcar geometry, the phase of the complex 2DES spectrum was approximately calibrated by comparing its projected spectrum onto the probe frequency axis with the corresponding pump-probe spectrum measured independently. In stark contrast, not only the phase of the SM-2DES spectrum but also the probe pulse spectrum can be corrected by using the interferometrically measured pump-probe spectrum (IPS) with precisely the same experimental setup.[1]

Let us consider the IPS signal field of which spectrum is denoted as $E_{PP}(\omega_2, T_w)$ and 2DES field with its spectrum to be $E_{2D}(\omega_2, T_w, \omega_1)$. Unlike the conventional heterodyne-detected pump-probe spectroscopy, we could measure both the real and imaginary parts of $E_{PP}(\omega_2, T_w)$ simultaneously. Thus, we can use both the amplitude and phase spectra of $E_{PP}(\omega_2, T_w)$ to achieve the phasing correction of the 2DES spectrum. First, $E_{PP}(\omega_2, T_w)$ can be easily normalized using the probe spectrum ($E_0(\omega_2)$) measured independently. 2DES can be similarly expressed as a normalized form

by introducing two complex scaling factors ($\alpha(\omega_2)$ and $\beta(\omega_2)$). $\alpha(\omega_2)E_0(\omega_2)$ represents the effective probe spectrum in the SM-2DES measurements. $\beta(\omega_2)$ is introduced here to correct the amplitude and phase of the 2DES signal. Thus, using the normalized forms of the IPS and the 2DES, the corresponding phase factors can be written as

$$\exp\left[i\Phi_{PP}\left(\omega_2,T_w\right)\right]=\frac{E_0\left(\omega_2\right)+E_{PP}\left(\omega_2,T_w\right)}{E_0\left(\omega_2\right)} \tag{41}$$

$$\exp\left[i\Phi_{2D}\left(\omega_2,T_w\right)\right]=\frac{\alpha\left(\omega_2\right)E_0\left(\omega_2\right)+\beta\left(\omega_2\right)E_{2D}\left(\omega_2,T_w\right)}{\alpha\left(\omega_2\right)E_0\left(\omega_2\right)}, \tag{42}$$

$$\text{where}\quad E_{2D}\left(\omega_2,T_w\right)\equiv\int d\omega_1 E_{2D}\left(\omega_2,T_w,\omega_1\right). \tag{43}$$

As can be seen in the definition of $E_{2D}(\omega_2, T_w)$, $E_{2D}(\omega_2, T_w)$ is the projected spectrum of $E_{2D}(\omega_2,T_w,\omega_1)$ onto the ω_2 axis. The phase angle spectrum, $\Phi_{PP}(\omega_2,T_w)$, is an experimentally measurable quantity because both $E_0(\omega_2)$ and $E_{PP}(\omega_2, T_w)$ were experimentally measured. Also, the projected spectrum, $E_{2D}(\omega_2, T_w)$, is obtained from our experimentally measured $E_{2D}(\omega_2, T_w, \omega_1)$, which is the sum of the rephasing and non-rephasing 2DES signals when T_w is sufficiently longer than the pulse duration time.[1] From the fact that the two phase-angle spectra, $\Phi_{PP}(\omega_2, T_w)$ and $\Phi_{2D}(\omega_2, T_w)$, must be the same, we have

$$\exp\left[i\Phi_{PP}\left(\omega_2,T_w\right)\right]-1=\frac{\beta\left(\omega_2\right)E_{2D}\left(\omega_2,T_w\right)}{\alpha\left(\omega_2\right)E_0\left(\omega_2\right)}$$

$$\equiv F\left(\omega_2\right)E_{2D}\left(\omega_2,T_w\right). \tag{44}$$

The left-hand side of Supplementary Equation 44 is entirely determined using experimentally measured $E_0(\omega_2)$ and complex $E_{PP}(\omega_2, T_w)$. Also, we have $E_{2D}(\omega_2, T_w)$ on the right-hand side of Eq.**오류! 참조 원본을 찾을 수 없습니다.**. Thus, once $F(\omega_2)$ is obtained using Supplementary Equation 44, it can be directly used to achieve normalization of amplitude and correction of the phase of SM-2DES spectra.

In the present work, the two rate-determining steps in the data processing of SM-2DES are (1) reading the recorded data onto the data processing space and (2) removing the slowly decaying components from the time-domain data $I(\omega_1, T_w, \omega_2)$ by carrying out a nonlinear least-square fitting

analysis. The latter was needed to carry out the Fourier transform of $I(\omega_1, T_w, \omega_2)$ with respect to T_w, which results in the coherent vibrational spectrum at (ω_1, ω_2) in the 2D frequency space. It took about 4 min to average 48 data sets and about 1 min for Fourier-transforming the averaged data. However, we anticipate that such data analysis time can be significantly shortened by using a more efficient numerical analysis method in the future.

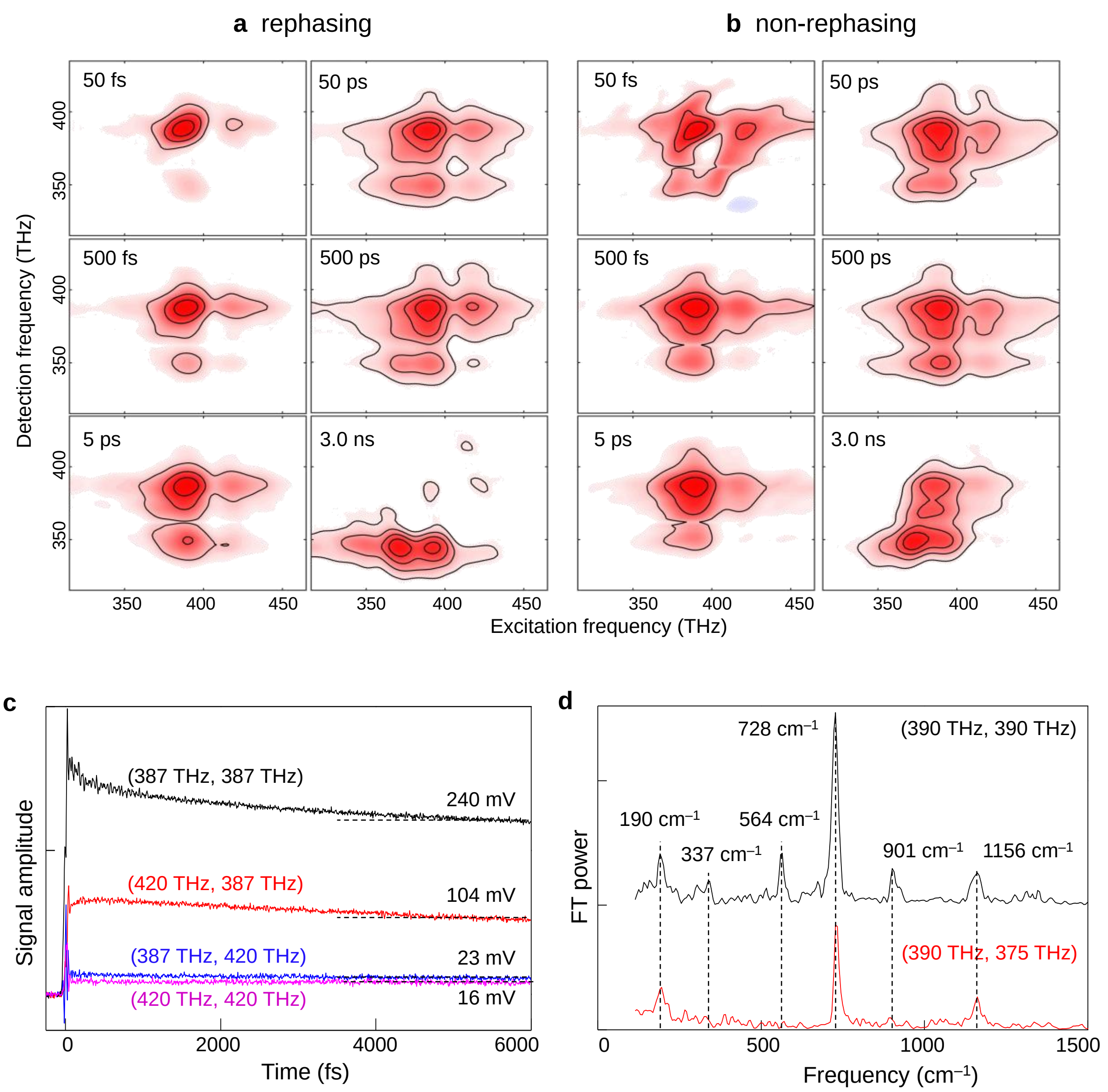

Supplementary Figure 2| Supplementary 2DES data of BChla 1-propanol solution. a-b, Rephasing (a) and non-rephasing (b) SM-2DES spectra of BChla 1-propanol solution at T_w = 50 fs, 500 fs, and 5 ps (Δf_r = 38.4 Hz), and at T_w = 50 ps, 500 ps, and 3 ns (Δf_r = 3.2 kHz). **c**, The total (rephasing + non-rephasing) absorptive 2DES time-profiles of BChla 1-propanol solution at (387 THz, 387 THz) (black, GSB_0 in the main text), (420 THz, 387 THz) (red, GSB_1 in the main text), (387 THz, 420 THz) (blue), and (420 THz, 420 THz) (violet). Each marked number in mV indicates the amplitude of each 2DES signal at 6000 fs. **d**, The coherent vibrational spectra extracted from the total (rephasing + non-rephasing) absorptive 2DES signals at (390 THz, 390 THz) (black), (390 THz, 375 THz) (red), where the two points are the maximum amplitude points of the 2D spectra of PC_1 and PC_2 in Fig. 4. It should be emphasized that the time step of 6 fs and the scanning time up to 6 ps for the T_w-scan data in S2c, obtained with using an ASOPS scheme, are very accurate because the automatic time-delay generation is controlled by locking the repetition frequencies to the atomic clock-generated reference frequency. Therefore, the frequencies of vibronically coupled modes (in Fig. R5 and S2d) do not require any frequency calibration.

Supplementary Note 4: Thermal grating effect

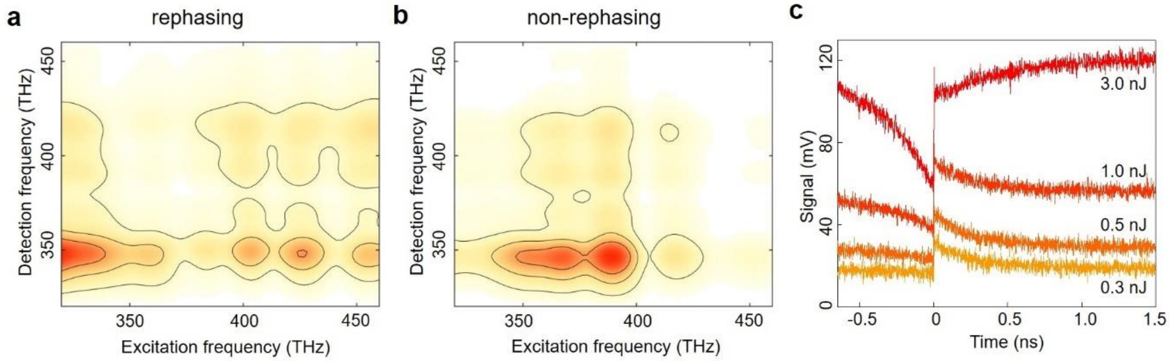

Supplementary Figure 3| Contribution of the thermal grating to the SM-2DES signal. a,(**b**,) The rephasing (non-rephasing) SM-2DES signal of IR125 ethanol solution at T_w < 0 with the probe pulse energy of 0.5 nJ. **c**. The T_w-scan data at τ_1 = 0 for varying probe pulse energy.

In 2DES experiments, transient grating generated by an interferometric excitation of chromophores by two non-collinearly propagating pump pulses disappears in time due to population relaxation of the excited state. The electronic transition energy is then converted into heat, increasing local temperature around chromophores. If such an excitation-dissipation process is repeated rapidly, the system won't have sufficient time to relax to a thermal equilibrium state. In the present SM-2DES, we use high repetition rate MLs (f_r = 80 MHz). Due to the non-collinear geometry, where the two pump and one probe pulses all propagate in different directions, a thermal grating is created, which then undesirably contributes to the 2DES spectrum. In fact, such thermal grating contribution was ignored entirely in the conventional 2DES experiments mainly because the Ti:Sapphire laser used has a repetition rate of 1 kHz, so it was safe to assume that the thermal grating is fully relaxed and disappeared before the next pump interaction after 1 ms. This is not the case for our SM-2DES utilizing 80 MHz MLs. If the thermal diffusion is much slower than the repetition rate, the system will reach a new quasi-equilibrium state, so the thermal grating does not interfere with the 2DES data, and instead, it provides a phase reference as described in Supplementary Note 3. Supplementary Figure 2a and b show the rephasing and non-rephasing SM-2DES signal at T_w = 12.5 ns –500 fs, respectively, where the thermal grating contribution is predominant. In particular, the broad spectral features along both excitation and detection frequencies of the two 2D spectra indicate that they originate from an electronically non-resonant response of the sample. However, if the population relaxation of a given photochemical system is

dominated by nonradiative processes, the SM-2DES can be of limited use because the thermal grating formed could diffract the incident light beams.

To further confirm that the thermal grating contribution to the SM-2DES signal cannot be ignored when high repetition rate lasers with sufficiently strong power, we additionally carried out 2DES measurements for varying probe pulse energy (Supplementary Figure 3c). In principle, both the thermal grating and the electronically resonant response signals should increase together with the probe intensity. Indeed when the probe pulse energy is relatively low, e.g., 0.3 and 0.5 nJ, both the background level and the decay component in the T_w-scan data, which correspond to the thermal grating and the transient grating signal, respectively, linearly depend on the probe pulse energy. However, if the probe pulse energy is high, e.g., 1.0 and 3.0 nJ, the background signal at around $T_w = 0$ shows a negative dip, which makes the numerical analysis of data difficult. Since the pump pulse energies are the same at a fixed value when we obtain the four different data shown in Supplementary Figure 3c, the negative dip at around $T_w = 0$ when the probe energy is high cannot originate from the difference in pump pulse energy. Thus, the unexpected and undesired negative feature in the background signal arises from a probe-induced effect. To minimize it, we lowered the probe power down to 0.6 nJ when we carried out SM-2DES measurements with ΔT_w = 500 fs. Fortunately, even with slightly high power probe pulses (1.2 nJ), the background signal remains constant for almost several picoseconds. Therefore, when we carried out SM-2DES measurements with $\Delta T_w = 6$ fs, we used probe pulses whose energy was 1.2 nJ.

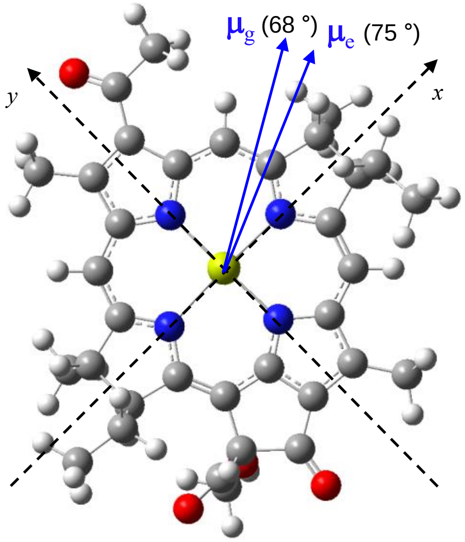

Supplementary Figure 4| Electric dipole moment of BChla. The electric dipole moments of BChla in S_0 (μ_g) and Q_y (μ_e) states are represented by blue arrows. The numbers in parentheses are the angles between the *y*-axis and the electric dipole moments.

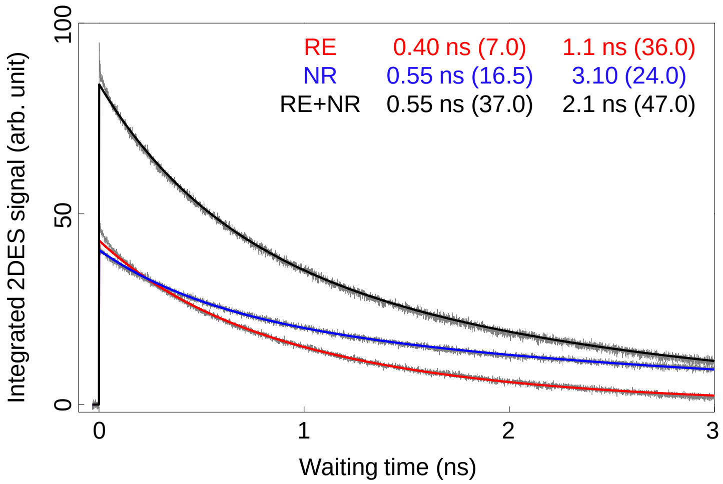

Supplementary Figure 5| Slow decay components in BChla. The grey curves represent the integrated rephasing (RE), non-rephasing (NR), and total (RE+NR) 2DES signals. The bi-exponential fitting results of RE (red), NR (blue), and total (black) signals are overlaid on the raw data. The fitting parameters are given in the upper part of this figure, where the numbers in the parentheses represent the amplitude of the relevant decay components. The fitting window in this nonlinear least-square fitting is from 0.25 ns to 3.0 ns.

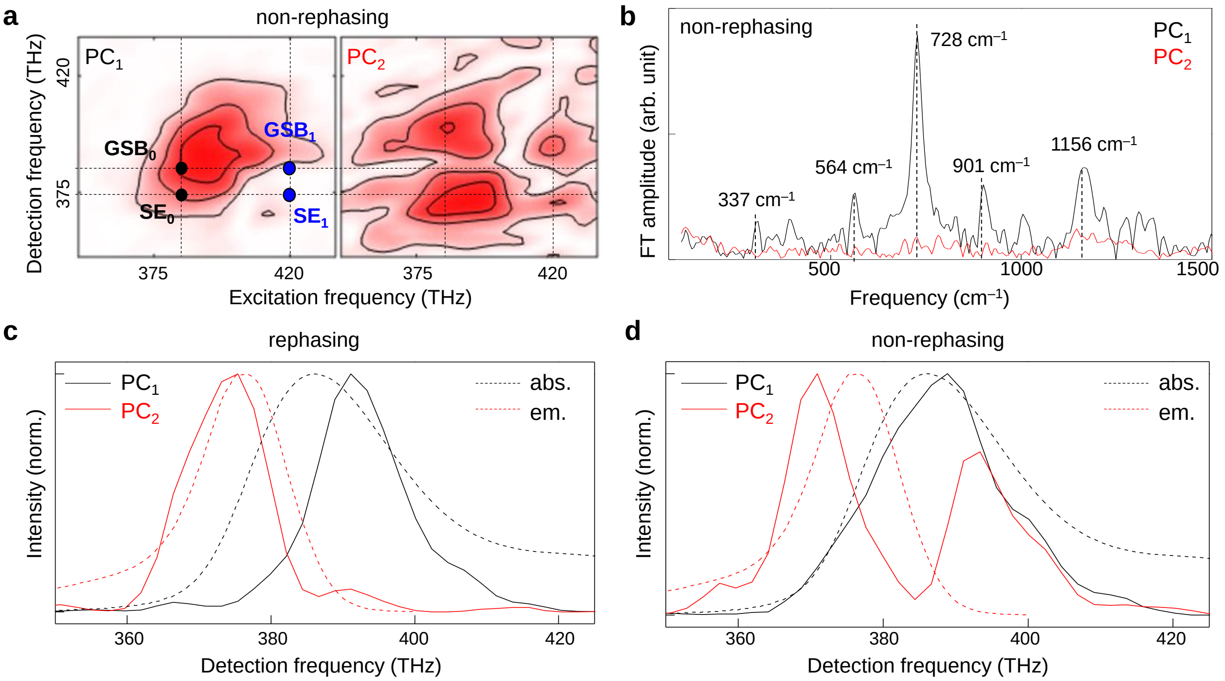

Supplementary Figure 6| Non-rephasing 2D coherent vibrational spectra of BChla. a, The 2D optical frequency spectra of two major principal components (PC_1 and PC_2) of the 2D coherent vibrational spectra for non-rephasing interaction pathway. **b,** The vibrational spectra of (**a**) multiplied by their score. Relatively intense five peaks at 337, 564, 728, 901, and 1156 cm^{-1} are marked with their centre frequencies. **c-d,** The 2D electronic spectra of PC_1 (black) and PC_2 (red) integrated over excitation frequency (< 400 THz) for rephasing (**c**) and non-rephasing (**d**) interaction pathways. The absorption (black, dashed line) and emission (red dashed line) spectra of BChla 1-propanol solution are overlaid for comparison.

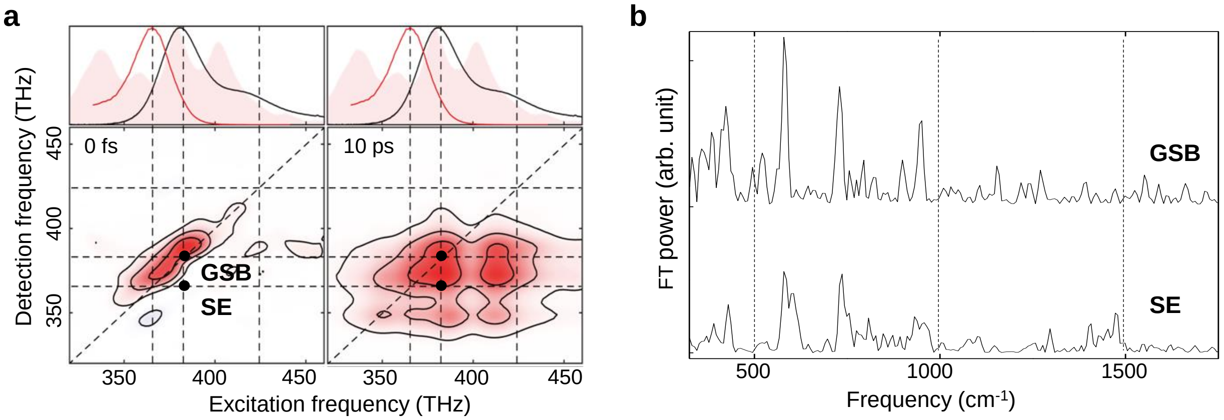

Supplementary Figure 7| Coherent vibrational spectrum of IR125/ethanol solution. a. Rephasing SM-2DES spectra of IR125/ethanol solution at $T_w = 0$ ($\Delta f_r = 32$ Hz) and 10 ps ($\Delta f_r = 1.6$ kHz). The absorption (black) and emission (red) spectra of IR125 in ethanol are shown in the top panels. In addition, the power spectrum of the light source (pink area) is presented for the sake of direct comparison. **b**. Coherent vibrational spectra of IR125 at the stimulated emission (SE) and ground-state bleach (GSB), which are marked by black circles in (**a**).

Supplementary Note 5: Gated sampling to protect samples from photochemical damage

Chlorophyll molecules, including BChla, are easily oxidized in the atmosphere and damaged by the photo-induced local heat. In conventional 2DES experiments with low repetition rate lasers, the chlorophyll solution is degassed before the measurement, and its 2DES signal is measured with a rotating cell for room temperature measurement. However, the rotating cell, which usually provides hundreds of rotations per second, is not recommended for SM-2DES with high-repetition-rate MLs due to its slow refresh rate.

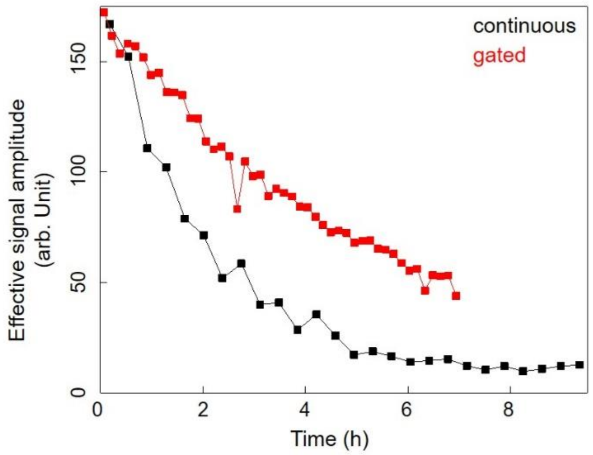

Supplementary Figure 8| The effect of gated sampling for the SM-2DES measurement of BChla/1-propanol solution at room temperature. The effective amplitude of the 2DES signal with continuous (black) and gated (red) sampling as a function of measurement time.

In this research, we flowed the BChla solution with a gear pump, which provides a pulsation-less liquid circulation, and gated sampling described in Supplementary Note 1 to minimize the photo-thermal damage of BChla molecules. We also chilled the gear pump with a circulating chiller to reduce the heat generated at the pump. The SM-2DES signal disappears in 5 min under the absence of the pump-chilling system. Supplementary Figure 3 shows the amplitude of the SM-2DES signal as a function of laboratory time. Since the decay of the 2DES signal corresponds to the BChla degradation, it can be confirmed that the use of gated sampling (red in Supplementary Figure 8) extends the lifetime of BChla by three times. Although it is impossible to remove the photochemical damage and to prevent the sample reservoir from being exposed to air, the SM-2DES can measure tens of data set before the degradation effect becomes dominant. Among the 46 data sets measured with the gated sampling, only 33 data sets were averaged because we lose signal-to-noise ration with more than 33 data sets.

Supplementary Note 6: Coherent vibrational spectrum simulation

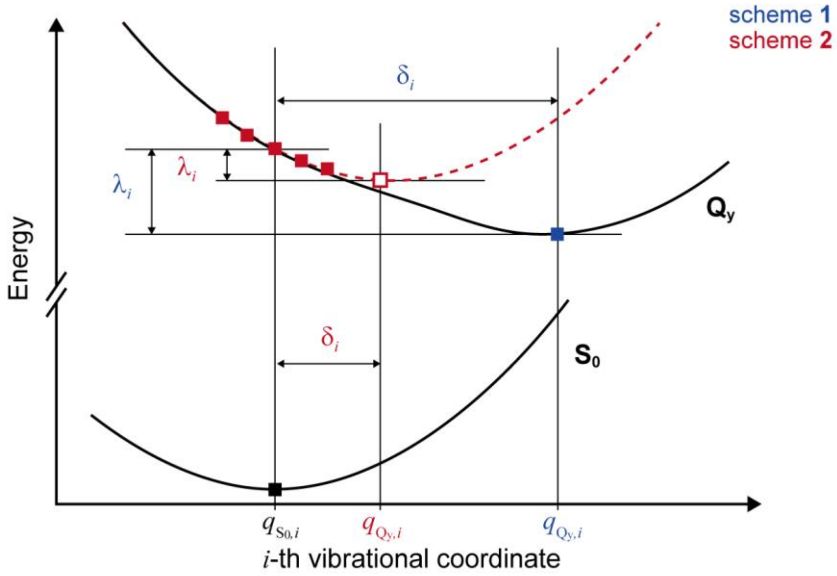

Supplementary Figure 9| Coherent vibrational spectrum simulation. The schematic diagram for the two coherent vibrational spectrum simulation schemes. $q_{A,i}$ indicates the equilibrium position of the electronic state "A" of the *i*th vibrational coordinate. δ_i and λi are the displacement and vibrational reorganization energy of *i*-th mode, respectively. Blue (red) coloured parameters are utilized for scheme **1** (scheme **2**) introduced in the main text. The filled squares indicate the quantum mechanically calculated points, while the empty square does the point estimated by extrapolation in scheme **2**.

The amplitude of a coherently oscillating vibrational mode activated by an impulsive photoexcitation, which corresponds to the vibronic coupling strength of the vibrational mode, is determined by the potential energy surface (PES) difference between the electronic ground and excited states involved in the relevant transition. Within the harmonic approximation with respect to the PES along all vibrational coordinate of the molecular system, the coupling strength of each vibrational mode can be simply estimated by calculating the geometrical displacement between the two electronic states along the vibrational coordinate. Therefore, once we know the equilibrium structure of the ground state ($\boldsymbol{q}_{S0}$) and its vibrational mode, it is possible to estimate the initial time-evolution of the excited molecule. In this research, we employed two types of simulations (scheme **1** and **2** in the main text) to assign the vibrational peaks in the coherent vibrational spectrum (CVS), which is the Fourier transform of the nuclear wave packet oscillation observed in time-resolved spectroscopy, observed in the SM-2DES data of BChla.

The two schemes require the geometrical difference between the equilibrium structures of the S_0 and Q_y states in terms of vibrational coordinates because the amplitude of each mode in CVS is

proportional to its vibrational reorganization energy ($\lambda_i = h\nu\delta_i^2/2$). Supplementary Figure 8 illustrates how the two schemes calculate the vibrational displacement (δ_i) between two equilibrium positions ($\boldsymbol{q}_{S0,i}$ and $\boldsymbol{q}_{Qy,i}$) on a vibrational coordinate. The difference between the two schemes is how to estimate the equilibrium structure of Q_y state ($\boldsymbol{q}_{Qy}$). In scheme **1**, $\boldsymbol{q}_{Qy}$ is directly calculated with a quantum chemical calculation and represents the structural difference as the linear combination of vibrational coordinates using the normal-mode projection method.[7] In scheme **2**, we calculated five single-point energies of the Q_y state by varying the displacement of the vibrational modes of the S_0 state. Based on the five points, the minimum energy structure of the Q_y state can be estimated by solving the quadratic equation.

If the actual PESs of a molecule satisfy the harmonic approximation, the calculation results from schemes **1** and **2** should be the same. In general, however, the harmonic approximation is only applicable for the initial state (S_0 in this research), but not for the final state (Q_y in this research) as illustrated in Supplementary Figure 9. In case the equilibrium structural difference between the two electronic states is significant, scheme **1** will provide more accurate information on the excited state dynamics, while scheme **2** is appropriate for the negligible difference. Although we employed these methods just for peak assignment due to the large size of BChla, these methods are quite accurate enough for small chromophores to investigate the molecular dynamics of the excited state.[8]

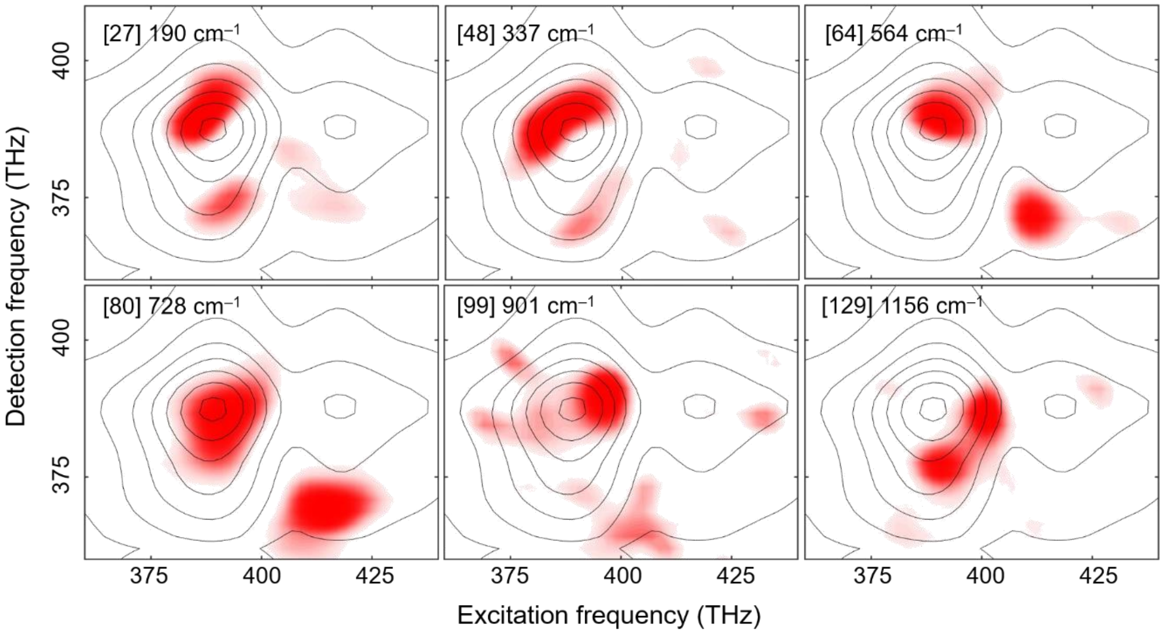

Supplementary Figure 10| Rephasing 2D vibrational coherence maps of BChla. 2D vibrational coherence maps of BChla at 190, 337, 564, 728, 901, and 1156 cm^{-1} extracted from the corresponding rephasing 2DES data. The number in each square bracket indicates the normal mode index, and the contour line represents the rephasing 2DES spectrum at 2 ps.

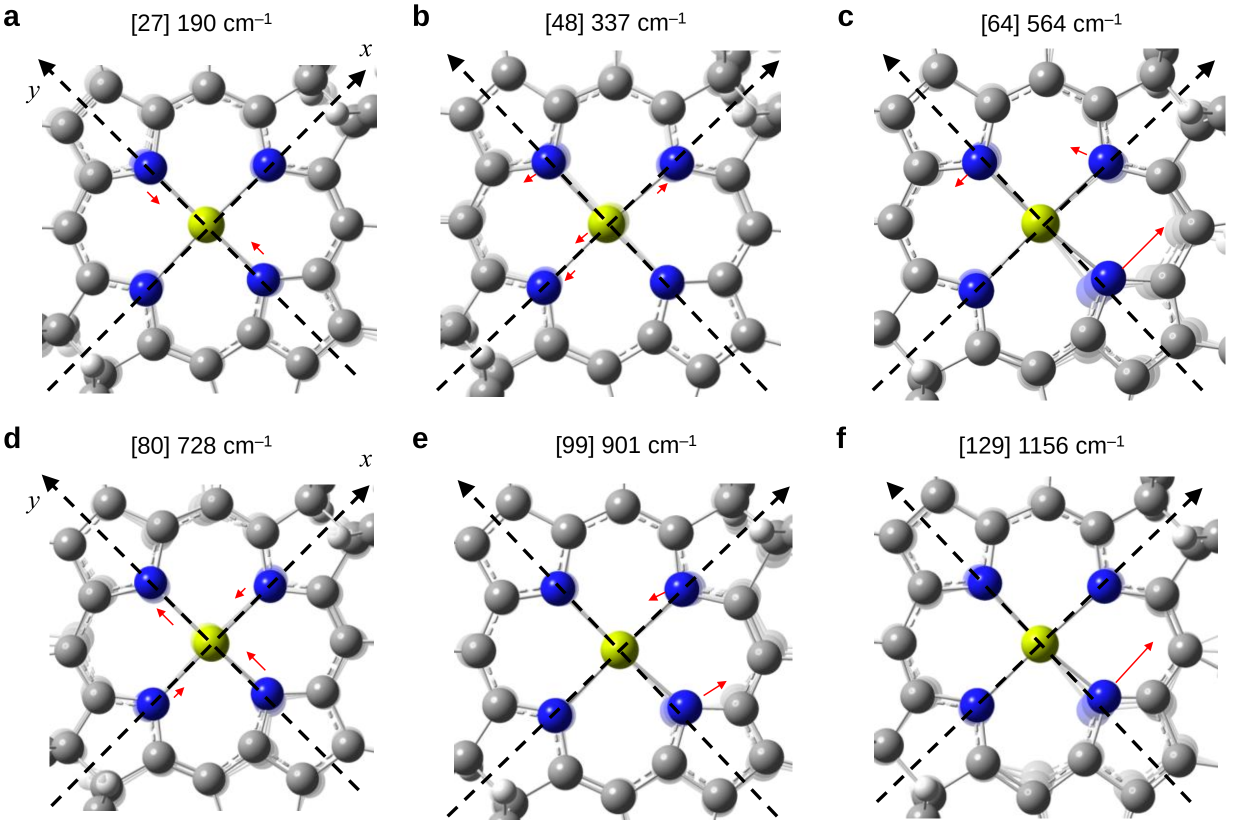

Supplementary Figure 11| Vibrational eigenvectors of six modes that are strongly coupled to the Q_y transition. The number in each square bracket indicates the normal mode index. The two dashed arrows are the molecular *x*- and *y*-axes (see Fig. 5). The red arrows represent the eigenvector elements.

SUPPLEMENTARY REFERENCES

1. Kim J, Yoon TH, Cho M. Interferometric measurement of transient absorption and refraction spectra with dual-frequency comb. *J. Phys. Chem. B* **122**, 9775-9785 (2018).
2. Niedzwiedzki DM, Blankenship RE. Singlet and triplet excited state properties of natural chlorophylls and bacteriochlorophylls. *Photosynth. Res.* **106**, 227-238 (2010).
3. Cundiff ST. Phase stabilization of ultrashort optical pulses. *J. Phys. D* **35**, R43 (2002).
4. Jeon J, Kim J, Yoon TH, Cho M. Dual frequency comb photon echo spectroscopy. *J. Opt. Soc. Am. B* **36**, 223-234 (2019).
5. Jeon J, Kim J, Yoon TH, Cho M. Theory of three-pulse photon echo spectroscopy with dual frequency combs. *J. Opt. Soc. Am. B* **36**, 3196-3208 (2019).
6. Cho M. *Two-dimensional Optical Spectroscopy*. CRC Press (2009).
7. Reimers JR. A practical method for the use of curvilinear coordinates in calculations of normal-mode-projected displacements and Duschinsky rotation matrices for large molecules. *J. Chem. Phys.* **115**, 9103-9109 (2001).
8. Kim J, Kim DE, Joo T. Excited-state dynamics of Thioflavin T: planar stable intermediate revealed by nuclear wave packet spectroscopies. *J. Phys. Chem. A* **122**, 1283-1290 (2018).
